# Validity and Reliability of a Smart Band for Monitoring Cardiorespiratory Parameters in Children and Adolescents with Severe Cerebral Palsy

**DOI:** 10.3390/s26030828

**Published:** 2026-01-27

**Authors:** Angélica Guerrero-Blázquez, Ángela Concepción Álvarez-Melcón, José Javier López-Marcos, Patricia Martín-Casas, Adrián Arranz-Escudero, Rosa María Ortiz-Gutiérrez

**Affiliations:** 1 Doctoral Program in Healthcare, Faculty of Nursing, Physiotherapy and Podiatry, Universidad Complutense de Madrid, Plaza de Ramón y Cajal, s/n, Ciudad Universitaria, 28040 Madrid, Spain; angeli06@ucm.es (A.G.-B.); adarranz@ucm.es (A.A.-E.); 2 Department of Physiotherapy, Faculty of Nursing, Physiotherapy and Podiatry, Universidad Complutense de Madrid, Plaza de Ramón y Cajal, s/n, Ciudad Universitaria, 28040 Madrid, Spain; josejalo@ucm.es (J.J.L.-M.); pmcasas@enf.ucm.es (P.M.-C.); rosaorti@ucm.es (R.M.O.-G.); 3 Comprehensive Integral Rehabilitation, Physiotherapy and Neurorehabilitation Research Group, Universidad Complutense de Madrid, Plaza de Ramón y Cajal, s/n, Ciudad Universitaria, 28040 Madrid, Spain; 4 InPhysio Research Group, Health Research Institute of the Hospital Clínico San Carlos (IdISSC), C/ Prof Martín Lagos, s/n, 28040 Madrid, Spain

**Keywords:** cerebral palsy, heart rate, oxygen saturation, wearable electronic devices

## Abstract

Cerebral palsy (CP) is a disorder frequently associated with respiratory and cardiac comorbidities, making the monitoring of heart rate (HR) and oxygen saturation (SpO_2_) essential. This study examined the reliability and validity of Xiaomi Mi Band 6, compared with a clinical pulse oximeter, for measuring HR and SpO_2_ in 35 children and adolescents with CP classified at GMFCS levels III–V. Mi Band 6 demonstrated good reliability for HR (ICC = 0.83), although the high measurement error (MDC_90_ = 19.57 bpm) limits its usefulness for small physiological changes. SpO_2_ results showed low reliability (ICC = 0.55) and substantial variability (MDC_90_ = 18.85%), exceeding the clinically acceptable error margin of ±2–3%. Validity analyses revealed poor agreement between Mi Band 6 and clinical pulse oximeter for SpO_2_, and moderate agreement for HR, with large variability in Bland–Altman analyses. Factors such involuntary movements, altered muscle tone, low body weight, and reflective sensors on the wrist may have affected the results. In conclusion, Xiaomi Mi Band 6 demonstrated good reliability and may be cautiously used for general HR monitoring, but it is not suitable for assessing SpO_2_ in this pediatric population. Further research is needed to identify cost-effective and accurate wearable technologies.

## 1. Introduction

Cerebral palsy (CP) encompasses a group of permanent disorders of posture and movement development, resulting from a non-progressive lesion of the central nervous system during fetal development or in the immature brain. Individuals with CP constitute a heterogeneous group due to differences in the nature and location of the brain injury, variability in the type and severity of associated symptoms, the degree of functional capacity, and concurrent impairments. This heterogeneity gives rise to distinct CP subtypes, each with varying impacts on quality of life. Quality of life has been shown to be associated with functional level and disability, being lower in individuals classified at higher levels of the Gross Motor Function Classification System (GMFCS) [1].

Beyond the motor, cognitive, and behavioral symptoms that characterize CP, affected individuals are also prone to comorbidities. Among these, cardiac and respiratory impairments represent the leading causes of morbidity and mortality. Such impairments are primarily associated with ineffective airway clearance, deformities of the spine and chest wall, respiratory muscle weakness, and recurrent respiratory infections. Respiratory dysfunction is particularly prevalent among children and adolescents with CP at higher GMFCS levels, especially those with profound functional limitations [2]. Compared with typically developing peers, children with CP exhibit elevated resting heart rate (HR) and reduced HR variability. The lowest HR variability is observed in those with the greatest functional impairment [3]. Consequently, the monitoring of vital signs is essential for guiding interventions and evaluating their effects in the clinical management of children and adolescents with CP at GMFCS levels III and IV.

Monitoring respiratory status involves recording oxygen saturation (SpO_2_) and heart rate (HR) parameters [4]. Electrocardiography is considered the gold standard for HR monitoring; however, its application in children and adolescents with CP poses challenges due to postural limitations, poor selective motor control, and concomitant difficulties related to perception, cognition, communication, and behavior. Pulse oximeters are the most widely used devices in both clinical and home settings for recording SpO_2_ and HR [5]. These devices are based on photoplethysmography (PPG), an optical technique that detects changes in peripheral blood volume. Nevertheless, their use in pediatric populations with CP and severe functional limitations depends on the child’s muscle tone and tolerance to pressure on the finger or hand.

The commercialization of wearable sensors has led to the development of a wide range of affordable, noninvasive, and ergonomic products capable of monitoring vital physiological parameters. Specifically, smart bands using PPG technology have emerged as an alternative to conventional pulse oximeters. Smart bands allow measurements to be obtained in a more natural manner, minimizing baseline interferences. These accessible, portable, and low-cost devices enable continuous monitoring of cardiorespiratory function both in clinical settings and at home [6].

The results of previous studies have demonstrated a high level of accuracy and agreement between smart bands and gold-standard instrumental systems for recording respiratory rate (RR) in adult and geriatric populations [7]. However, these devices have not yet received regulatory approval for clinical application as health monitoring tools, and therefore cannot be marketed as medical devices [8]. It is thus necessary to validate the fitness for purpose of this technology.

Although wearable sensors have been extensively studied in adult populations, their application in pediatric groups remains a highly relevant area of ongoing research [9]. To date, no studies have been found that evaluated the use of smart bands to monitor children and adolescents with cardiorespiratory compromise. Heart rate and oxygen saturation monitoring are essential for assessing functional status and detecting adverse events in clinical populations. Consumer-grade photoplethysmography-based devices offer a low-cost and accessible alternative; however, their accuracy may be affected by factors such as movement, peripheral perfusion, and anatomical characteristics. This issue is particularly relevant in children and adolescents with cerebral palsy (CP), whose clinical and anthropometric variability may limit the reliability of wearable measurements. Therefore, validation against clinically approved devices is necessary to determine their accuracy and potential clinical applicability. For this reason, we propose a validation and reliability study of a smart band for recording SpO_2_ and HR in children and adolescents with CP at GMFCS levels III–V.

## 2. Materials and Methods

### 2.1. Ethical Statement

The study protocol was approved by the Ethics Committee of Hospital Clínico San Carlos, Madrid, Spain (code 22/732-P_EC). The legal guardians of all participants provided written informed consent prior to inclusion, in accordance with the Declaration of Helsinki (revised 2013). Patient privacy and data confidentiality were ensured throughout the study.

### 2.2. Sample

Participants were recruited by convenience sampling from the María Soriano Special Education Center (Madrid, Spain). Eligible participants were children and adolescents with CP at GMFCS levels III, IV, and V, aged between 3 and 18 years, who were enrolled at the center. Exclusion criteria were school absenteeism lasting more than two consecutive months due to health problems.

### 2.3. Devices

In this study, the Mi Band 6 (Xiaomi, Beijing, China) was selected as a representative commercial smart band due to its wide availability and accessibility, and because the sensing technologies it incorporates for HR and SpO_2_ measurement are comparable to those implemented in most consumer-grade smart bands currently available on the market. Therefore, the findings of this study are likely to be applicable to a broad range of similar wrist-worn wearable devices using reflective PPG. In addition, the device was chosen based on the following practical criteria: (1) suitability for relatively small wrists to ensure proper fit for all participants; (2) comfort for daily wear; (3) long battery life to avoid daily recharging; and (4) commercial availability and a price compatible with the project budget.

HR and SpO_2_ are measured at the dorsal side of the wrist using reflective PPG technology. The system consists of LED light emitters—green for HR and red and infrared for SpO_2_—whose light is internally reflected within the skin and dermis. The amount of reflected light varies depending on local blood flow. Photodetectors positioned adjacent to the emitters capture the returning light. For HR detection, the oscillation in reflected light intensity corresponds to each cardiac cycle. In the case of SpO_2_, the device compares the levels of reflected red and infrared light to estimate blood SpO_2_, based on the differential absorption characteristics of oxygenated and deoxygenated hemoglobin (oxygenated hemoglobin absorbs more infrared and less red light, whereas deoxygenated hemoglobin shows the opposite pattern) [10,11]. The values from the smart band are shown on a mobile phone application: Zepp Life mobile application for Android (Anhui Huami Information Technology Co., Ltd., Hefei, China), available at: https://play.google.com/store/apps/details?id=com.huami.watch.hmwatchmanager (accessed on 16 January 2023).To verify the accuracy of HR and SpO_2_ measurements, the finger pulse oximeter Nellcor™ Bedside SpO_2_ (Medtronic, Dublin, Ireland) was used as the gold standard. This pulse oximeter is a clinically validated hospital-grade device widely used for continuous monitoring of SpO_2_ and HR. This device is based on transmissive PPG system consisting of red and infrared LED light emitters on one side and photodetectors on the opposite side. The device is placed on the distal phalanx of the index finger, where detectors record light absorption by non-pulsatile tissues (direct current component) and reflect the rhythmic changes in arterial blood volume with each heartbeat (alternating current component), which allow estimation HR. The ratio between both currents for each wavelength (red and infrared) allows for the estimation of SpO_2_ [10,11]. The HR and SpO_2_ values from the pulse oximeter are displayed on its digital screen.

The pulse oximeter does not represent the absolute gold standard (such as arterial blood gas analysis for SpO_2_ or ECG for HR); however, its use is widely accepted in pediatric clinical settings and represents the most clinically appropriate and feasible non-invasive reference method for measuring heart rate and oxygen saturation in this population with severe functional impairments [12,13]. This choice was motivated by ethical and practical considerations in children with severe CP, who have significant limitations regarding invasive tests or bulky equipment.

### 2.4. Data Collection

The outcome variables were HR, measured in beats per minute (bpm), and SpO_2_, expressed as the percentage of oxygen saturation in blood. Variables were recorded simultaneously using the Mi Band 6 (placed on the left wrist) and the pulse oximeter (placed on the index finger of the right hand). During data collection, participants remained seated in their usual wheelchair in a relaxed position.

The smartband was placed on the dorsal aspect of the forearm, approximately one or two fingerbreadths above the wrist, ensuring firm contact with the skin to prevent device slippage. In participants with structural flexion deformities, the device was positioned just proximal to these areas to avoid excessive pressure that could restrict blood flow.

After device placement, both devices were activated and allowed to complete their internal adjustment process, as the sensor required a few seconds to collect sufficient data to calculate heart rate and display the result.

Each participant was monitored for 150 s, and the values were recorded at the end of this period. Values were only accepted if SpO_2_ and HR remained stable for a minimum interval of 5–10 s, without large oscillations or changes in sensor location. Episodes of rapid and marked transient drops in SpO_2_ that resolved within seconds and coincided with agitation or handling of the child were classified as motion artifacts, and data recording was postponed until these events had ceased.

Data were collected from all participants at three different times: Moment 1 (M1), upon arrival at school; Moment 2 (M2), during the morning break before lunch; and Moment 3 (M3), at the end of the school day before returning home. At each time point, three consecutive recordings of each variable were obtained.

To characterize the sample, health data were collected from medical records and through interviews with the participants’ legal guardians, conducted by the principal investigator.

### 2.5. Statistical Analysis

All analyses were performed using Jamovi software version 2.3 (2022) based on R software. (The jamovi project, Sydney, Australia) [14]. A descriptive analysis of all study variables was conducted, including mean values, standard deviations (SD), and 95% confidence intervals (CI).

To assess intra-device reliability of both the pulse oximeter and the smart band, the intraclass correlation coefficient (ICC) was calculated using an average, two-way mixed-effects model with absolute agreement, based on measurements obtained during Moment 1. Reliability was classified as follows: excellent (ICC ≥ 0.90), good (0.90 > ICC ≥ 0.70), fair (0.70 > ICC ≥ 0.40), and poor (ICC < 0.40) [15]. The standard error of measurement (SEM) was calculated using the formula SD × √(1 − ICC) [16]. The minimal detectable change at the 90% confidence level (MDC90) was computed using the formula SEM × √2 × 1.65 [17].

To evaluate the validity of HR and SpO_2_ values obtained from the Mi Band 6 compared with the reference pulse oximeter, agreement between the two devices was primarily assessed using Bland–Altman analysis. The range of ±1.96 SD from the mean difference was established as the 95% limits of agreement (LoA) [18]. In parallel, the ICC was calculated using a single-measure, two-way mixed-effects model with absolute agreement. Given that SpO_2_ is a bounded variable (0–100%) and that values in this sample were clustered near the upper limit, the normality assumptions required for parametric reliability statistics may be violated. Therefore, in addition to the ICC, non-parametric Spearman’s rank correlation coefficients were calculated.

## 3. Results

### 3.1. Descriptive Analysis

A total of 35 children meeting the inclusion criteria participated in the study. The mean age was 13.5 ± 2.9 years, and the mean BMI was 18.0 ± 6.3. Most participants were classified as GMFCS level V (65.7%). The characteristics of the study sample are summarized in Table 1.

### 3.2. Reliability Analysis

The intra-device reliability analysis revealed marked differences between the reference pulse oximeter and the smart band for the outcome variables. For SpO_2_, the pulse oximeter demonstrated good reliability, with an ICC of 0.86 (95% CI: 0.75–0.92). In contrast, the smart band exhibited moderate to low reliability, with an ICC of 0.55 (95% CI: 0.21–0.75). The SEM was substantially higher for the smart band (8.08%) compared with the pulse oximeter (1.05%), resulting in MDC_90_ values of 18.85% and 2.45%, respectively. These findings indicate that the smart band does not provide sufficiently consistent SpO_2_ measurements to detect clinically relevant changes in this population.

For HR, both devices showed higher reliability. The pulse oximeter achieved excellent reliability, with an ICC of 0.97 (95% CI: 0.95–0.98), a SEM of 0.41 bpm, and an MDC_90_ of 0.95 bpm. The smart band demonstrated good reliability, with an ICC of 0.83 (95% CI: 0.71–0.91). Nevertheless, its SEM was 8.39 bpm and the MDC_90_ reached 19.57 bpm, suggesting that only changes exceeding this threshold could be interpreted as true physiological variations rather than measurement error.

The results of the reliability analysis are presented in Table 2.

### 3.3. Validity Analysis

The Bland–Altman analysis for SpO_2_ showed small mean differences between devices at M1 (MD = 0.63%) and M2 (MD = 0.57%) indicating minimal systematic bias. This variability was more pronounced at M3 (MD: 1.42%). However, the limits of agreement (LoA) were wide at all time points (M1: −6.78 to 7.34%; M2: −5.35 to 6.50%; M3: −3.01 to 5.81%), reflecting considerable variability in individual measurements, limiting the utility of the smart band for accurate SpO_2_ assessment under these conditions.

The validity analysis using ICC revealed poor agreement between the smart band and the reference pulse oximeter across all three measurement points. The ICC values were 0.30 (95% CI: 0.03–0.57) at M1, 0.31 (95% CI: 0.02–0.57) at M2, and 0.01 (95% CI: −0.33–0.32) at M3, indicating a limited capacity of the smart band to accurately estimate SpO_2_ in this pediatric CP population. At M1, the Spearman correlation between devices was weak and not statistically significant (Rho = 0.27, *p* = 0.11). Similarly, at M2, a weak, non-significant correlation was observed (Rho = 0.24, *p* = 0.17). In contrast, at M3, the correlation increased to a moderate level and reached statistical significance (Rho = 0.49, *p* = 0.001). Despite the statistically significant correlation observed at M3, the overall strength of association across measurement moments remained low to moderate, indicating limited concordance between the smart band and the reference device for SpO_2_ assessment. These results support that the Xiaomi Mi Band 6 is not suitable for reliable SpO_2_ monitoring in children and adolescents with severe CP. A sensitivity analysis excluding an extreme SpO_2_ value from the smart band observed at M3 was performed. Although the limits of agreement narrowed slightly, they remained well beyond clinically acceptable thresholds, and the overall conclusions regarding poor agreement were unchanged.

For HR, the smart band consistently underestimated values compared with the pulse oximeter. This underestimation was more pronounced at M1 (MD = 5.34 bpm; LoA: −18.39 to 29.07) and M3 (MD = 5.40 bpm; LoA: −23.04 to 33.84), while it was smaller at M2 (MD = 2.91 bpm; LoA: −14.75 to 20.58). Although the mean differences were relatively modest, the LoA ranged from −23.04 to 33.84 bpm, corresponding to an error margin of up to ±30 bpm. These findings highlight a high degree of variability in individual HR measurements, which limits the clinical applicability of the smart band for a parameter that requires high measurement accuracy.

The ICC values for HR indicated a more acceptable level of agreement. At M1, moderate agreement was observed (ICC = 0.74; 95% CI: 0.54–0.85), which was similar at M3 (ICC = 0.58; 95% CI: 0.31–0.76), while good agreement was recorded at M2 (ICC = 0.76; 95% CI: 0.58–0.87). These findings suggest that, despite certain limitations, the smart band demonstrated greater validity for HR measurement than for SpO_2_ in this population. In the same line, Spearman’s correlation revealed stronger and statistically significant associations between devices. At M1 a moderate correlation was observed (rho = 0.45, *p* = 0.00), which increased to a strong correlation at M2 (rho = 0.75, *p* < 0.001) and M3 (rho = 0.59, *p* < 0.001). The results of the validity analysis are summarized in Table 3. 

**Figure 1:**
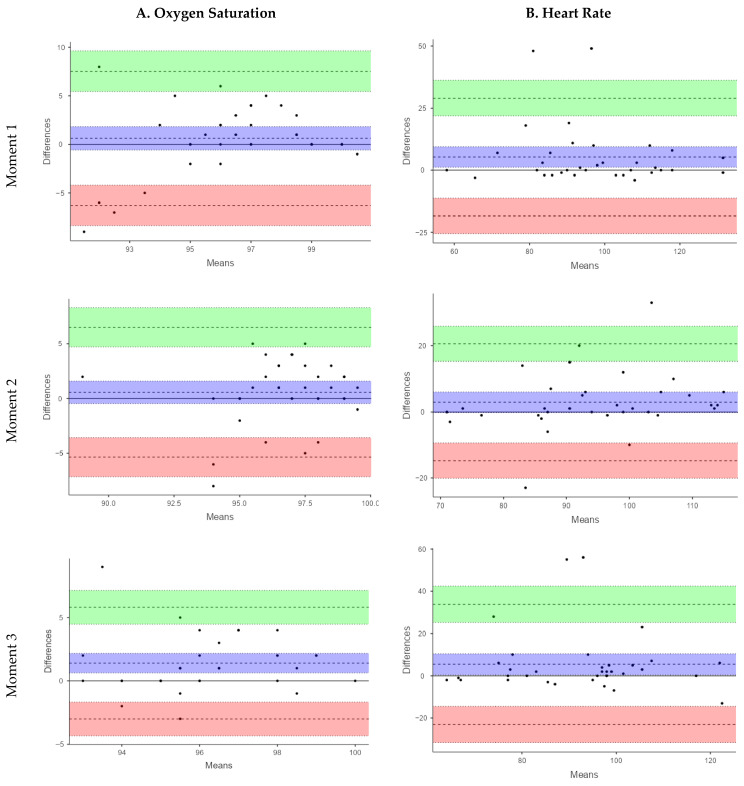
Bland–Altman plots of relative agreement between devices for SpO_2_ and HR data.

Figure 1 presents the Bland–Altman plots. In these plots, the central line represents the mean difference (bias) between devices, while the upper and lower lines indicate the 95% limits of agreement. Although small mean differences suggest minimal systematic bias, the wide limits of agreement observed—particularly for SpO_2_—indicate high inter-individual variability. This level of variability implies that the two devices cannot be considered interchangeable for clinical use.

## 4. Discussion

In the present study, we evaluated the validity and reliability of a commercial smart band for measuring HR and SpO_2_ in a pediatric population with CP classified between levels III and V of the GMFCS.

With respect to SpO_2_ measurement, the results showed low intra-device reliability for the smart band, as evidenced by a high SEM of 8.08% and an MDC_90_ of 18.85%. These values substantially exceed the clinically acceptable accuracy typically reported for pulse oximeters under controlled test conditions (±2% to ±3%) and the approximate clinical limits of agreement observed in real-world settings (~±4%), thereby limiting the utility of the Mi Band 6 for monitoring clinically relevant physiological changes [19,20].

Similar findings have previously been reported in studies of other low-cost alternative devices, such as home-use pediatric pulse oximeters and mobile applications, when compared with clinical pulse oximeters with specifications similar to those used in the present study [21].

In contrast, the clinical pulse oximeter used as the reference demonstrated greater reliability and consistency in SpO_2_ measurements, with an SEM of 1.05% and an MDC_90_ of 2.45%. Nevertheless, it is important to note that previous research has reported inaccuracies in these devices when SpO_2_ values are at or below 85% in pediatric populations [22]. In our study, mean SpO_2_ values across the three measurement points were above this threshold, which may have contributed to the greater stability observed in the reference pulse oximeter measurements.

Regarding HR, both devices demonstrated good levels of reliability. The pulse oximeter showed excellent reliability, with low SEM and MDC_90_ values, supporting its suitability as a clinical reference. The smart band demonstrated good reliability; however, the SEM value (8.39 bpm) exceeds the established error threshold of ≤±5 bpm, which is considered a clinically acceptable variability parameter for HR monitors [23]. Regarding the MDC_90_ (19.57 bpm), changes smaller than this threshold may be attributed solely to measurement inaccuracies of the smart band (e.g., noise, motion artifacts, reading errors) and not necessarily to a true modification in HR.

With respect to the validity of the Xiaomi Mi Band 6, both the ICC and the Bland–Altman analyses for SpO_2_ indicated low validity and poor agreement with the reference pulse oximeter. Although the mean differences between devices were small, the wide LoA (±7%) reflected high individual variability.

This discrepancy was even greater at M3, where the mean difference initially tripled and the limits of agreement widened to ±30%. Therefore, a sensitivity analysis excluding an extreme SpO_2_ value from the smart band observed at M3 was performed. The extreme SpO_2_ value is likely attributable to transient peripheral perfusion artifacts rather than true hypoxemia, as values at earlier moments were within normal ranges. Importantly, exclusion of this outlier did not materially alter the study conclusions.

The interpretation of ICC values for SpO_2_ should be made with caution, as SpO_2_ is a bounded variable subject to ceiling effects. Such distributional characteristics may violate the assumptions underlying variance-based reliability coefficients and potentially bias ICC estimates. However, complementary non-parametric analyses and Bland–Altman agreement plots consistently demonstrated wide limits of agreement and substantial measurement error, supporting the conclusion that the smart band is not suitable for reliable SpO_2_ assessment in this population.

The high intra-device variability, the occurrence of extreme values, and the wide limits of agreement observed suggest that SpO_2_ assessment with the smart band is compromised in this population. These findings are likely driven by population-specific factors associated with severe CP, including frequent involuntary movements (particularly those involving the finger extensor musculature), marked alterations in muscle tone, and anthropometric characteristics such as reduced wrist circumference, which impair adequate device fit [24]. Together, these conditions degrade both mechanical sensor stability and peripheral cutaneous perfusion at the wrist, thereby compromising the physiological and technological assumptions required for reliable PPG estimation of SpO_2_.

In contrast, HR measurements showed greater stability and agreement with the clinical reference device, although the limits of agreement were also wide (±30 bpm). This variability was higher than that reported in studies with healthy individuals (±11.5 bpm) [25] and with individuals with severe motor disabilities (±16 bpm) [26], in which wrist-based PPG devices and chest monitors were used during sedentary activities. In our population, this variability may be related to dysfunctions in autonomic cardiac modulation, which are more pronounced at higher GMFCS levels (IV and V) [3].

The differences observed between the smart band and the clinical pulse oximeter may also be attributed to technological factors and the anatomical location of the sensors. Blood perfusion, which is critical for accurate HR and SpO_2_ measurement, is greater in the distal areas of the fingers, where the clinical pulse oximeter is placed, compared with the dorsal side of the wrist, where the smart band sensors make contact. Common conditions in children with CP, such as vasoconstriction, increased muscle tone, and fatigue, may negatively affect the optical signal of PPG sensors, thereby reducing measurement quality [27].

Among the parameters influencing the PPG signal, previous studies have identified body mass index, skin tone, body temperature, and factors related to sensor positioning, including sensor location, contact pressure, and particularly motion artifacts. These aspects should be taken into account in future research through the development of standardized measurement protocols to improve the feasibility of using PPG sensors in clinical settings [28].

Although both devices used in the present study (smart band and clinical pulse oximeter) employ PPG technology, they differ in their processing algorithms. The smart band uses a reflective sensor, which is more susceptible to interference from superficial bony structures and reduced muscle thickness at the wrist. This factor may have influenced measurement accuracy, particularly in the 51.4% of participants who were underweight. In this regard, Li et al. have reported that transmissive sensors provide greater stability than reflective ones [29].

To overcome the limitations observed in SpO_2_ monitoring, future research should focus on alternative wearable approaches better suited to pediatric populations with severe motor impairments. This includes the use of wearable devices incorporating transmissive rather than reflective PPG sensors, which may improve signal stability and SpO_2_ accuracy in populations with compromised peripheral perfusion. Another relevant aspect is the development of signal-processing algorithms trained on motor-impaired populations, incorporating adaptive motion-artifact rejection and low-perfusion compensation models. Moreover, future wearable technologies should integrate real-time signal quality indices to identify unreliable recordings. With regard to the physical design of the device, signal quality could be improved through optimized band fit and ergonomic placement, promoting consistent skin contact.

This study has several limitations. Data collection at a single center restricted the sample size and imposed a fixed daily schedule, which may have influenced the physiological parameters assessed, particularly toward the end of the day. Nevertheless, this routine facilitated a calm and comfortable environment favorable for data acquisition. Future multicenter studies would allow for larger sample sizes and enable exploration of the validity of these devices in contexts with greater functional demands. It would also be necessary to evaluate other models and brands of smart bands available on the market, including those incorporating electrocardiography (ECG) technology, which may provide greater accuracy than PPG. In this sense, a further limitation of this study is the absence of ECG, which remains the gold standard for HR measurement. Although the clinical pulse oximeter used as a reference is widely accepted in pediatric practice, it relies on PPG and is therefore also susceptible to motion artifacts and perfusion-related errors. In addition, the signal-processing and artifact-rejection algorithms implemented in commercial smart bands are proprietary and not publicly disclosed, precluding direct comparison between devices. It has been suggested that smart band algorithms may be optimized using adult training datasets and may incorporate smoothing or regression toward adult mean HR values, which could partially explain the systematic differences observed in this pediatric population with severe motor impairments. Finally, it should be noted that although validity was assessed at three different time points, intra-device reliability was calculated based on a single measurement, which can be considered a limitation, as physiological conditions and signal stability may vary throughout the day. Future research should include repeated measurements at different times of the day and under different physiological conditions to better capture temporal variability and strengthen the robustness of intra-device reliability estimates.

In conclusion, the findings of this study indicate that, although the smart band may be acceptable for general HR monitoring, it is not a valid or reliable device for assessing SpO_2_ in pediatric populations with CP and compromised functional capacity, given its high variability and low level of accuracy. In contexts where reliable monitoring and sensitivity to small physiological changes in HR and SpO_2_ are required, the use of this type of smart band is inappropriate.

## 5. Conclusions

The present study evaluated the validity and reliability of the Xiaomi Mi Band 6 smart band for monitoring HR and SpO_2_ in children and adolescents with CP at GMFCS levels III–V. The results demonstrated that, while the device provided acceptable reliability for HR monitoring, its performance for SpO_2_ was poor, with high variability and limited agreement compared with the clinical reference standard. These findings indicate that commercial smart bands incorporating reflective PPG sensors, exemplified in this study by Mi Band 6, are not suitable for accurate SpO_2_ assessment in this population, although they may have potential for general HR monitoring. Future studies with larger, multicenter samples and alternative wearable technologies are warranted to identify low-cost, clinically reliable tools for continuous cardiorespiratory monitoring in pediatric populations with severe functional limitations.

## Figures and Tables

**Table 1:** Characteristics of participants.

Variable	Mean ± SD	n (%)
Age (years)	13.5 ± 2.9	
Height (meter)	1.4 ± 0.2	
Weight (kilogram)	33.7 ± 17.1	
Sex		Male, 19 (54.3%)
		Women, 16 (45.7%)
BMI	18.0 ± 6.3	Underweight, 18 (51.4%)
		Healthy, 13 (37.1%)
		Overweight, 3 (8.6%)
		Obesity, 1 (2.9%)
GMFCS		Level III, 2 (5.7%)
		Level IV, 10 (28.6%)
		Level V, 23 (65.7%)

SD: standard deviation; BMI: body mass index; GMFCS: Gross Motor Function Classification System.

**Table 2:** Reliability intra-device.

	Device	ICC (95% CI)	SEM	MDC_90_
SpO_2_	Pulsi oximeter	0.86 (0.75 to 0.92)	1.05	2.45
	Smart band	0.55 (0.21 to 0.75)	8.08	18.85
HR	Pulsi oximeter	0.97 (0.95 to 0.98)	0.41	0.95
	Smart band	0.83 (0.71 to 0.91)	8.39	19.57

SpO_2_: oxygen saturation; HR: heart rate; ICC: intraclass correlation coefficient; 95% CI: 95% confidence interval; SEM: standard error measurement; MDC_90_: minimal detectable change to a 90% confidence interval.

**Table 3:** Correlation analysis and Bland–Altman analysis between devices.

	M	Device	Mean ± SD	MD (95% LoA)	ICC (95% CI)	Rho	*p*
SpO_2_	M 1	Pulsi oximeter	96.7 ± 3.38	0.63 (−6.78 to 7.34)	0.30 (0.03 to 0.57)	0.27	0.11
		Smart band	96.2 ± 2.54				
	M 2	Pulsi oximeter	97.0 ± 2.76	0.57 (−5.35 to 6.50)	0.31 (0.02 to 0.57)	0.24	0.17
		Smart band	96.5 ± 2.37				
	M 3	Pulsi oximeter	96.9 ± 2.13	1.42 (−3.01 to 5.81)	0.01 (0.33 to 0.32)	0.49	0.00
		Smart band	95.5 ± 2.05				
HR	M 1	Pulsi oximeter	99.5 ± 17.0	5.34 (−18.39 to 29.07)	0.74 (0.54 to 0.85)	0.45	0.01
		Smart band	94.2 ± 18.6				
	M 2	Pulsi oximeter	95.3 ± 14.4	2.91 (−14.75 to 20.58)	0.76 (0.58 to 0.87)	0.75	<0.00
		Smart band	92.4 ± 12.4				
	M 3	Pulsi oximeter	94.9 ± 16.13	5.40 (−23.04 to 33.84)	0.58 (0.31 to 0.76)	0.59	<0.00
		Smart band	90.5 ± 16.8				

SpO_2_: oxygen saturation; HR: heart rate; M: moment; SD: standard deviation; MD: mean of difference; LoA: limits of agreement; 95% CI: 95% confidence interval; ICC: intraclass correlation coefficient; Rho: Spearman′s correlation coefficient.

## Data Availability

The data that support the findings of this study are not publicly available because they contain confidential patient information. Access to the data may be considered upon reasonable request to the corresponding author and with approval from the institutional ethics committee.

## Abbreviations

The following abbreviations are used in this manuscript:

CP	Cerebral palsy
GMFCS	Gross Motor Function Classification System
HR	Heart rate
PPG	Photoplethysmography
RR	Respiratory rate
SpO_2_	Oxygen saturation
